# Supplementary material for: Fast raster-scan optoacoustic mesoscopy enables assessment of human melanoma microvasculature in vivo
Source: Nat Commun. 2022 May 19;13:2803. doi: 10.1038/s41467-022-30471-9 (PMC9120110; doi:10.1038/s41467-022-30471-9)
Supplement: Supplementary file 1 — Supplementary Information [file 41467_2022_30471_MOESM1_ESM.pdf]

Supplementary Information

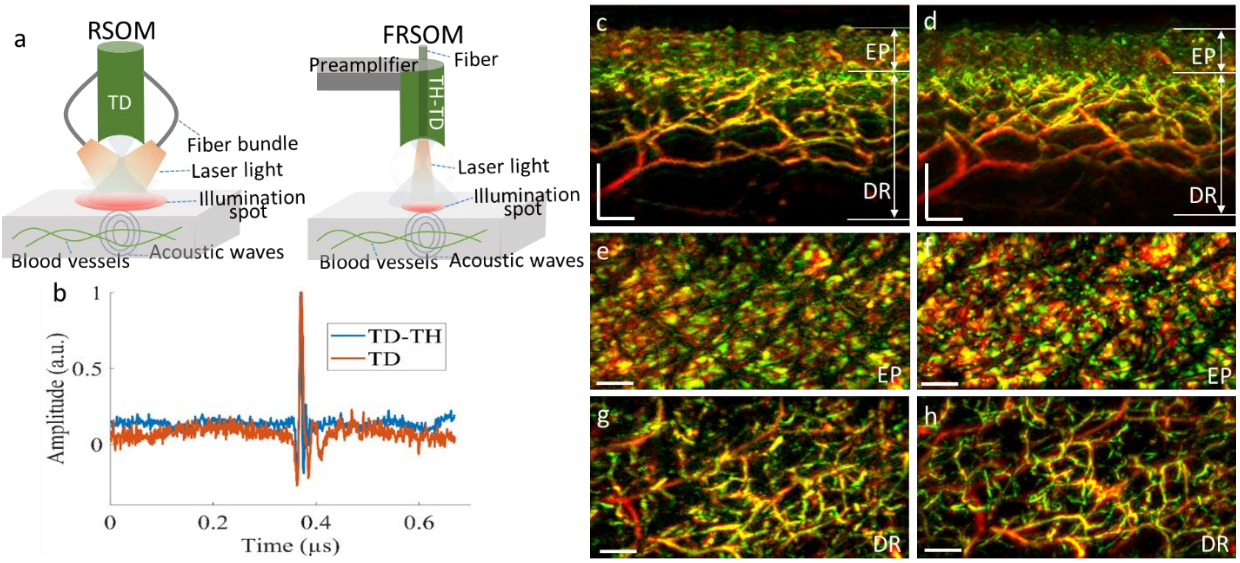

Fig. S1. Comparisons between the conventional RSOM and FRSOM. a. the schematic of the conventional RSOM and FRSOM. The conventional RSOM applied fiber bundle-based illumination with 2.5 mm diameter while the FRSOM employed the single fiber illumination with a 1.4 mm diameter. b. Signal quality comparisons between the TD and the TH-TD with preamplifier when measuring same object with external illumination. The TD-TH gains two times of SNR compared to the TD. c-d. Quality comparisons of the cross-sectional images between the conventional RSOM (c) and FRSOM (d) when measuring the same skin area at arm from a healthy volunteer (female). e-f and g-h are corresponding MIP images in the coronal directions from the epidermal (EP) and dermal (DR) layers respectively. We observed highly comparable image quality between the conventional RSOM and FRSOM. The healthy volunteer was measured multiple time with similar results. All scale bar: 500 µm.

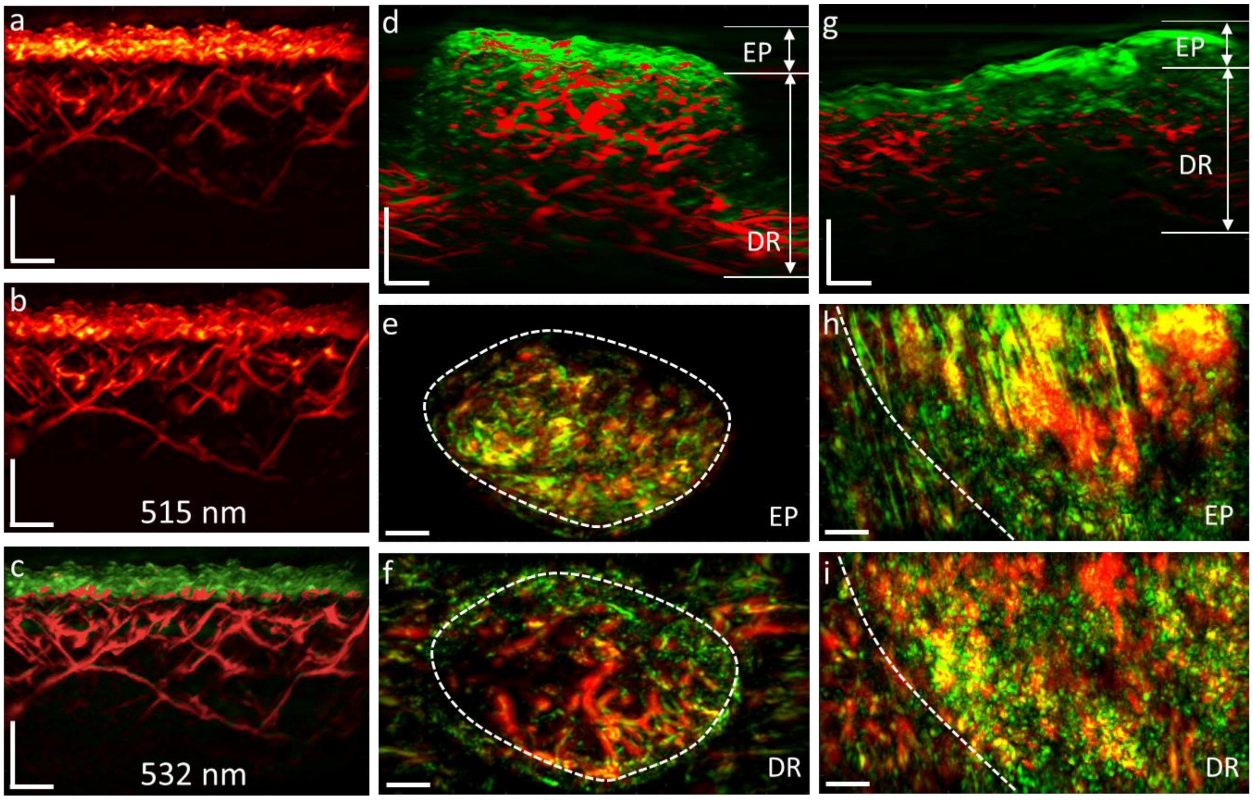

Fig. S2. The dual-wavelength FRSOM imaging. a. MIP image in the cross-sectional direction of healthy skin (male) recorded by the dual-wavelength FRSOM, where the reconstructed images were unmixed for melanin (green) and hemoglobin (red). b and c. MIP image at the cross-sectional direction recording by FRSOM at wavelength of 515 nm and 532 nm respectively. d. MIP image in the cross-sectional direction of a pigmented nevus (female) recorded by the dual-wavelength FRSOM, where the reconstructed images were unmixed for melanin (green) and hemoglobin (red). e and f. MIP spectra FRSOM image at the coronal direction of the nevus at the skin layers of epidermis (EP) and dermis (DR). The white dash lines indicate the pigmented lesion boundary. g. MIP image in the cross-sectional direction of a melanoma lesion nevus (female) recorded by the dual-wavelength FRSOM, where the reconstructed images were unmixed for melanin (green) and hemoglobin (red). h and i. MIP spectra FRSOM image at the coronal direction of the melanoma at the skin layers of epidermis (EP) and dermis (DR). The white dash lines indicate the pigmented lesion boundary. The dual-wavelength FRSOM successfully differentiates melanin and hemoglobin structures in the healthy skin, nevus and melanoma. The healthy skin of the volunteer, nevus and melanoma lesions were measured once. All scale bar: 500 µm.

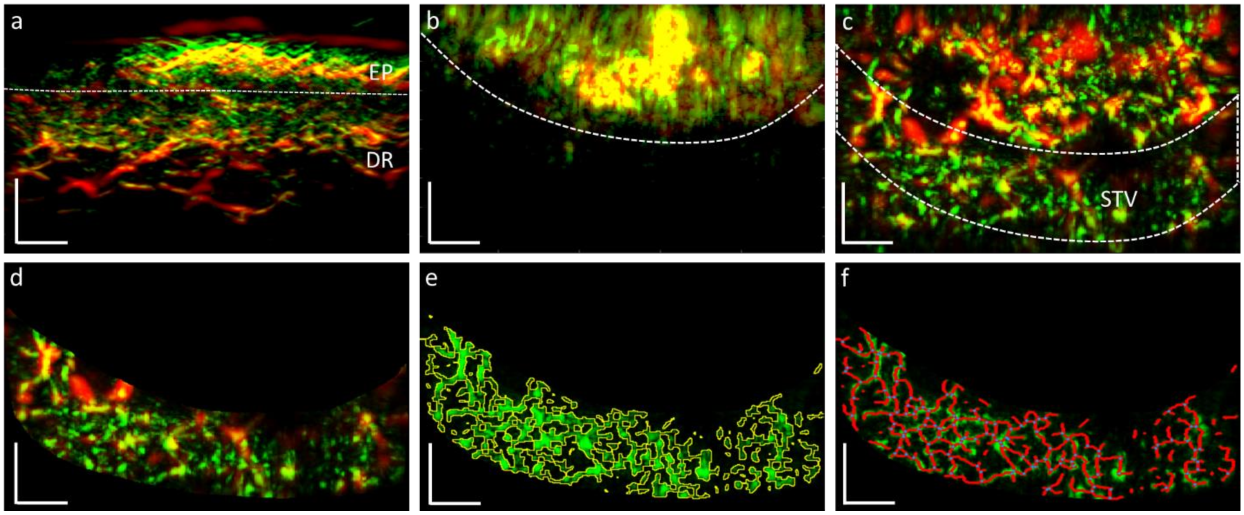

Fig. S3. The FRSOM image and vessel segmentation procedures. a. cross-sectional FRSOM image of a melanocytic lesion, where the epidermal (EP) and dermal (DR) layer is segmented by the white dash line. b. MIP image at the coronal direction corresponding to the epidermal layer of (a), where the dash line indicates the boundaries separating the pigmented area of the lesion and the surrounding skin tissue based on the melanin contrast. c. MIP image of dermal vasculature corresponding to the dermal layer of (a), where the surrounding tissue vessel (STV), segmented from the boundary line to 500 um thickness towards the healthy skin as indicated by the white dash box. d. the segmented image of the STV. e. the segmented vessel boundaries and the corresponding vessel centerlines (f). All scale bar: 500 μm.

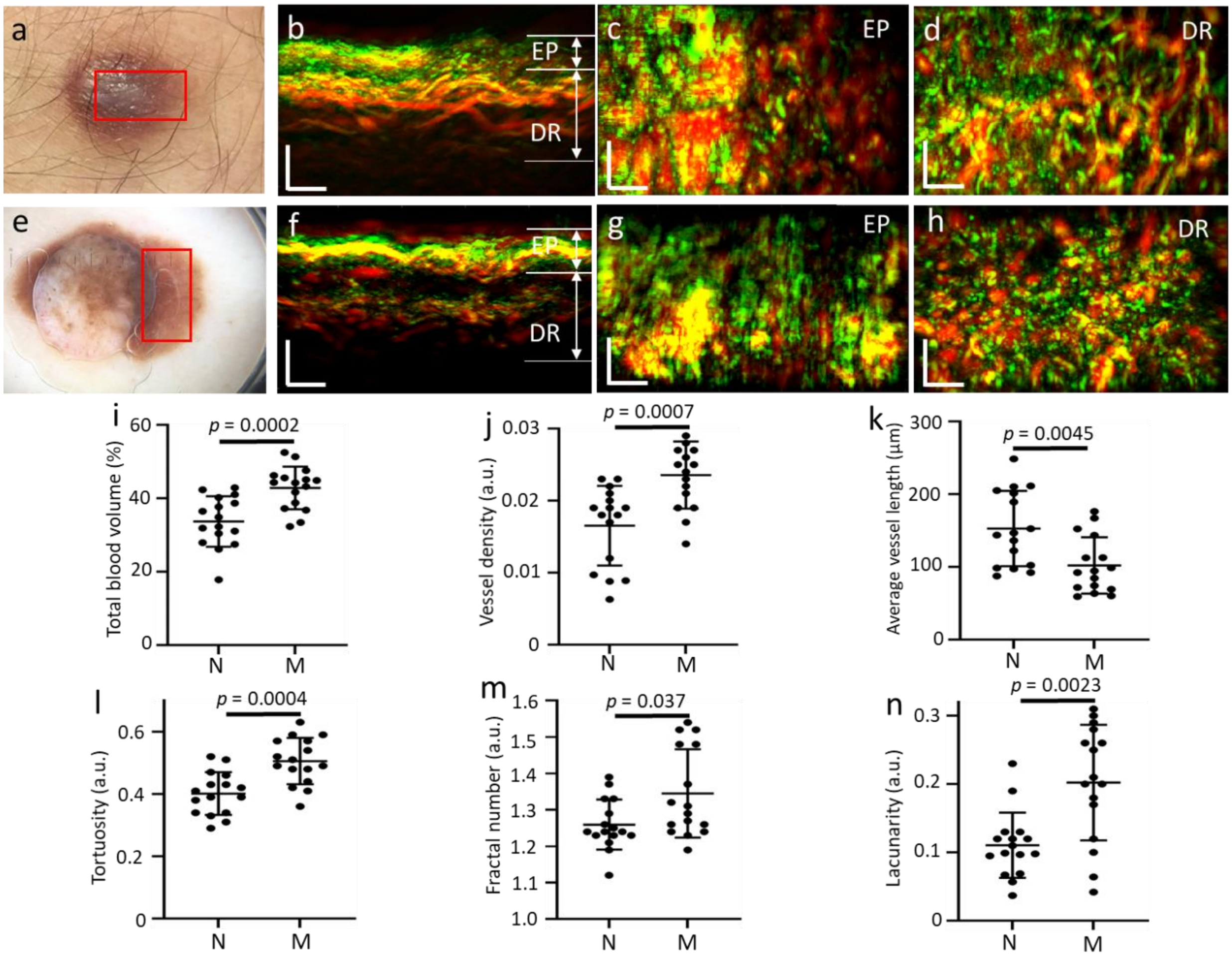

Fig. S4 Comparisons of the vasculature features acquired in the pigmented lesion center areas between nevi and melanomas groups. a, Photograph of a dysplastic nevus from a patient chest (male); the red rectangle indicates the scanning area. b, Cross-sectional MIP image measured at the center areas of the nevus marked by the red rectangle in (a). c,d, Corresponding MIP images in the coronal direction of the epidermis (EP) and dermis (DR) layers of (b). e, Photograph of a melanoma from a patient back (female). f, Cross-sectional MIP image measured at the center pigmented area of the melanoma marked by the red rectangle in (e). g,h, Corresponding MIP images in the coronal direction of the EP and DR layers of (f); i-n, the computed vessel biomarkers: total blood volume (i, 33.69% ± 6.88% vs. 42.82% ± 5.80%, p=0.0002), the vessel density (j, 0.017 ± 0.0055 vs. 0.024 ± 0.0046, p = 0.0007), average vessel length (k, 152.93 ± 51.72 vs. 102.17 ± 38.73, p = 0.0045), tortuosity (l, 0.41 ± 0.068 vs. 0.51 ± 0.074, p = 0.0004), fractal number (m, 1.26 ± 0.069 vs. 1.35 ± 0.12, p = 0.037) and lacunarity (n, 0.11 ± 0.048 vs. 0.20 ± 0.085, p = 0.0023) between the non-malignant nevi group (N, n=16) and melanoma group (M, n=16). All vessel biomarkers are computed from the MIP FRSOM images of the dermal vessels in the coronal direction. All patients have been measured once. Data are expressed as the mean ± SD. Statistical significance was calculated using two-tailed Mann–Whitney U tests. All patients was measured once. Source data are provided as a Source Data file. All scale bar: 500 μm.

Supplementary Table 1 Comparisons between conventional RSOM and FRSOM

System	Signal to noise ratio	Scanning speed (seconds)	Pulse lase energy (μJ)	Illumination spot size (mm^2)
Conventional RSOM	26.9	70	80	19.6
Fast RSOM (FRSOM)	55.1	15	18	6.15

Supplementary Table 2 characteristics of the patients with malignant melanoma (MM) and dysplastic nevus (DN)

Diagnosis	Age	Subtype	Location	Histology depth
MM	35	Nevus-associated melanoma	Upper back	0.3 mm
MM	78	SSM	Left elbow	1.3 mm
MM	78	SSM	Thigh	1.8 mm
MM	85	SSM	Upper back	0.7 mm
MM	75	LMM	Right cheek	0.2 mm
MM	82	NMM	Axilla	0.6 mm
MM	76	SSM	Upper arm	0.3 mm
MM	63	SSM	Shoulder	0.9 mm
MM	68	SSM	Upper arm	0.6 mm
MM	60	SSM	Forearm	1.4 mm
DN	60	AJN	Chest	0.9 mm
DN	73	AJN	Upper back	0.23 mm
DN	73	AJN	Chest	0.8 mm
DN	44	AJN	Thigh	0.3 mm
DN	53	AJN	Shoulder	0.4 mm
DN	50	AJN	Back	0.3 mm
DN	53	AJN	Shoulder	0.35 mm
DN	27	AJN	Axilla	1. 3 mm
CN	31	CN	Hand	0.42 mm
CN	21	CN	Foot	0.84 mm

Note: SSM, superficial spreading melanomas; LMM, lentigo maligna melanoma; NMM, nodular maligna melanoma; AJN, atypical junctional nevus, CN, Compound nevus
