# Supplementary material for: Fast raster-scan optoacoustic mesoscopy enables assessment of human melanoma microvasculature in vivo
Source: Nat Commun. 2022 May 19;13:2803. doi: 10.1038/s41467-022-30471-9 (PMC9120110; doi:10.1038/s41467-022-30471-9)
Supplement: Supplementary file 3 — Description of Additional Supplementary Information [file 41467_2022_30471_MOESM3_ESM.pdf]

Description of Additional Supplementary Information

File Name: Supplementary Movie 1

Description: FRSOM image volume acquired from a healthy volunteer. The upper part is the epidermis layer while the lower part is the dermal vasculature layer. The maximum intensity images (MIP) of the volume at different views are shown in Supplementary Fig. S1.

File Name: Supplementary Movie 2

Description: FRSOM image volume acquired from the center of a melanoma lesion. The scan region is indicated in Fig. 3 (Scan 1). The corresponding MIP images are shown in Fig. 3c,f,i. The z,x coordinates correspond to 4 mm × 2 mm, y is the depth direction.

File Name: Supplementary Movie 3

Description: FRSOM image volume acquired from the edge of a melanoma lesion. The scan region is indicated in Fig. 3 (Scan 2). The corresponding MIP images are shown in Fig. 3d,g,j. The z,x coordinates correspond to 4 mm × 2 mm, y is the depth direction.

File Name: Supplementary Movie 4

Description: FRSOM image volume acquired from the surrounding healthy skin of a melanoma lesion. The scan region is indicated in Fig. 3 (Scan 3). The corresponding MIP images are shown in Fig. 3e,h,k. The z,x coordinates correspond to 4 mm × 2 mm, y is the depth direction.

File Name: Supplementary Movie 5

Description: FRSOM image volume acquired from a nevus lesion of a volunteer. The upper part is the melanin signals from the epidermis layer while the lower part is the dermal vasculature layer.
