# Supplementary material for: Fast raster-scan optoacoustic mesoscopy enables assessment of human melanoma microvasculature in vivo
Source: Nat Commun. 2022 May 19;13:2803. doi: 10.1038/s41467-022-30471-9 (PMC9120110; doi:10.1038/s41467-022-30471-9)
Supplement: Supplementary file 9 — Reporting Summary [file 41467_2022_30471_MOESM9_ESM.pdf]

nature portfolio

Corresponding author(s): Vasilis Ntziachristos

Last updated by author(s): 2022.30.03

Reporting Summary

Nature Portfolio wishes to improve the reproducibility of the work that we publish. This form provides structure for consistency and transparency in reporting. For further information on Nature Portfolio policies, see our Editorial Policies and the Editorial Policy Checklist.

Statistics

For all statistical analyses, confirm that the following items are present in the figure legend, table legend, main text, or Methods section.

n/a	Confirmed	
[ ]	[x]	The exact sample size (*n*) for each experimental group/condition, given as a discrete number and unit of measurement
[ ]	[x]	A statement on whether measurements were taken from distinct samples or whether the same sample was measured repeatedly
[ ]	[x]	The statistical test(s) used AND whether they are one- or two-sided<br>*Only common tests should be described solely by name; describe more complex techniques in the Methods section.*
[ ]	[x]	A description of all covariates tested
[ ]	[x]	A description of any assumptions or corrections, such as tests of normality and adjustment for multiple comparisons
[ ]	[x]	A full description of the statistical parameters including central tendency (e.g. means) or other basic estimates (e.g. regression coefficient) AND variation (e.g. standard deviation) or associated estimates of uncertainty (e.g. confidence intervals)
[ ]	[x]	For null hypothesis testing, the test statistic (e.g. *F*, *t*, *r*) with confidence intervals, effect sizes, degrees of freedom and *P* value noted<br>*Give P values as exact values whenever suitable.*
[x]	[ ]	For Bayesian analysis, information on the choice of priors and Markov chain Monte Carlo settings
[x]	[ ]	For hierarchical and complex designs, identification of the appropriate level for tests and full reporting of outcomes
[x]	[ ]	Estimates of effect sizes (e.g. Cohen's *d*, Pearson's *r*), indicating how they were calculated

Our web collection on statistics for biologists contains articles on many of the points above.

Software and code

Policy information about availability of computer code

Data collection	The data acquisition was operated by a GAGE DAQ. The imaging system was controlled by custom-designed Matlab (2018) code.
Data analysis	The motion correction and image reconstruction algorithms are described in detail in the methods and our previous work as a reference. The image reconstruction code (back-projection method, cited) in the manuscript was accelerated with GPU acceleration. The main function of the image reconstruction code is shared in this public repository: https://doi.org/10.5281/zenodo.6466446. The image analysis methods are described with available methods from the literature as cited in the method section of the manuscript. Supplementary movies were generated by ImageJ. All statistical analyses were performed with GraphPad Prism 9.0 software (GraphPad Software Inc., San Diego, California, USA).

For manuscripts utilizing custom algorithms or software that are central to the research but not yet described in published literature, software must be made available to editors and reviewers. We strongly encourage code deposition in a community repository (e.g. GitHub). See the Nature Portfolio guidelines for submitting code & software for further information.

Data

Policy information about availability of data

All manuscripts must include a data availability statement. This statement should provide the following information, where applicable:
- Accession codes, unique identifiers, or web links for publicly available datasets
- A description of any restrictions on data availability
- For clinical datasets or third party data, please ensure that the statement adheres to our policy

All authors confirm that all data supporting the findings of the study are available. Raw optoacoustic data of all patients can be available upon reasonable request from the corresponding author, with permissionof the Klinik und Poliklinik für Dermatologie und Allergologie am Biederstein, Munich, Germany.

The raw data for characterizing the transducers and motion effects from healthy volunteers are shared in the public repository: https://doi.org/10.5281/zenodo.6466446. Source data are provided with this paper.

Field-specific reporting

Please select the one below that is the best fit for your research. If you are not sure, read the appropriate sections before making your selection.

☒ Life sciences ☐ Behavioural & social sciences ☐ Ecological, evolutionary & environmental sciences

For a reference copy of the document with all sections, see nature.com/documents/nr-reporting-summary-flat.pdf

Life sciences study design

All studies must disclose on these points even when the disclosure is negative.

Sample size	We measured two healthy volunteers and one patient with psoriasis. We acquired twenty lesions (10 melanomas from 10 patients and 10 nevi from 9 patients). In order to increase the sample size, 7 melanomas and 7 dysplastic nevi were measured two times at different edge areas of the lesion, generating 17 FRSOM datasets for the melanoma and nevus groups respectively. No sample size calculation was performed. The number of healthy volunteers and patients was chosen to sufficiently demonstrate the performance of our imaging system and the clinical potential as a pilot study.
Data exclusions	We have developed data quality control method as described in the method section, where lowquality datasets due to serious motion artefacts were excluded from the study.
Replication	The same imaging system have been used for all measurements. Similar imaging results have been obtained over multiple measurements with reported sample sizes (2 healthy volunteers and one patient with psoriasis), which show good reproducibility. All healthy volunteers and patients were measured independently and the data quality were evaluated based on the algorithms described in the method section. The imaging method has also been verified by our previous work as stated in the validation section in the methods. The data analysis methods have been fully validated as described in the methods as well.
Randomization	This is not relevant to our study, because we have two specific patient groups, patients with nevi and patients with melanoma. All lesions were diagnosed by professional dermatologists and pathologists based on the histology image.
Blinding	This is not relevant to our study, because we have two specific patient groups, patients with nevi and patients with melanoma. The selection of the nevus and melanoma areas to be imaged was made by professional dermatologists, independently of the authors that processed the data. All lesions were diagnosed by professional dermatologists and pathologists based on the histology image.

Reporting for specific materials, systems and methods

We require information from authors about some types of materials, experimental systems and methods used in many studies. Here, indicate whether each material, system or method listed is relevant to your study. If you are not sure if a list item applies to your research, read the appropriate section before selecting a response.

Materials & experimental systems

n/a	Involved in the study	
☒	☐	Antibodies
☒	☐	Eukaryotic cell lines
☒	☐	Palaeontology and archaeology
☒	☐	Animals and other organisms
☐	☒	Human research participants
☒	☐	Clinical data
☒	☐	Dual use research of concern

Methods

n/a	Involved in the study	
☒	☐	ChIP-seq
☒	☐	Flow cytometry
☒	☐	MRI-based neuroimaging

Human research participants

Policy information about studies involving human research participants

Population characteristics	We have measured a male (33 years old) and a female (31 years old) healthy volunteers and a patient (42 years old, male) with psoriasis. Twenty lesions (10 melanomas from 10 patients and 10 nevi from 9 patients) were imaged following approval from the Ethics Committee of Klinikum Rechts der Isar der Technischen Universität München, Munich, Germany. The melanoma patient group (6 female and 4 male) had an average age of 70.0 ± 14.6 years while the nevus group (4 female and 6 male) had an average of 48.5 ± 18.6 years. The measured lesions were located on the back, chest, upper shoulder, arm, and leg. The detailed characteristics of all patients are listed in supplementary Table II.All lesions were diagnosed by professional dermatologists and pathologists based on the histology image.
Recruitment	There was no self-selection. The healthy volunteers were randomly recruited to test the characterize system performance

Recruitment	between the conventional and the new imaging system, and analyze the effect of the breathing motion on the imaging quality. There is no bias and will not affect the system performance since the system has been fully characterized by multi measurements. Patients were recruited independently by clinical collaborators. Twenty lesions (10 melanomas from 10 patients and 10 nevi from 9 patients) were imaged following approval from the Ethics Committee of Klinikum Rechts der Isar der Technischen Universität München, Munich, Germany. All lesions were diagnosed by professional dermatologists and pathologists based on the histology image.
Ethics oversight	All participants gave written informed consent before the measurement. The Ethics Committee of Klinikum Rechts der Isar der Technischen Universität München, Munich, Germany

Note that full information on the approval of the study protocol must also be provided in the manuscript.
